# *MfWRKY40* Positively Regulates Drought Tolerance in *Arabidopsis thaliana* by Scavenging Reactive Oxygen Species

**DOI:** 10.3390/ijms26178495

**Published:** 2025-09-01

**Authors:** Xueli Zhang, Wei Duan, Yuxiang Wang, Zhihu Jiang, Qian Li

**Affiliations:** Key Laboratory of Ministry of Education of Grassland Resources and Ecology of Western Arid Region, Key Laboratory of Grassland Resources and Ecology of Xinjiang, College of Grassland Science, Xinjiang Agricultural University, Urumqi 830052, China; zzhangxl1010@163.com (X.Z.); dwcjatfx@163.com (W.D.); wyx9868@163.com (Y.W.); j15212051063@163.com (Z.J.)

**Keywords:** *Medicago falcata*, *Arabidopsis thaliana*, *MfWRKY40* gene, drought stress

## Abstract

Drought stress is a major abiotic constraint that severely restricts the growth of *Medicago falcata* L. by inducing the accumulation of reactive oxygen species (ROS) in plants. WRKY transcription factors (TFs) play a key role in regulating plant responses to drought stress. In this study, we investigated the role of the *MfWRKY40* gene in drought tolerance. Under mannitol and ABA stress treatments, *MfWRKY40*-overexpressing lines (OEs) showed significantly longer primary roots, increased lateral roots, and higher fresh weight compared to wild-type (Col) lines, indicating significantly enhanced growth and drought tolerance. Similarly, under soil drought conditions, transgenic *Arabidopsis thaliana* exhibited enhanced drought tolerance. NBT staining demonstrated decreased ROS accumulation in transgenic lines after stress treatment. Correspondingly, the *MfWRKY40*-overexpressing lines displayed significantly lower levels of hydrogen peroxide (H_2_O_2_), superoxide anion (O_2_^−^), and malondialdehyde (MDA) compared to Col, along with elevated activities of superoxide dismutase (SOD), catalase (CAT), and peroxidase (POD), as well as increased proline (Pro) content. Furthermore, *MfWRKY40* upregulated the expression of antioxidant enzyme genes (*AtPOD3*, *AtSOD4*, and *AtCAT1*) and modulated the expression of other drought-related genes. In summary, our results demonstrate that *MfWRKY40* enhances drought tolerance in *A. thaliana* by improving ROS scavenging capacity. This study provides a theoretical foundation for further exploration of *MfWRKY40*’s functional mechanisms in drought stress adaptation.

## 1. Introduction

During their normal life activities, plants are affected by various abiotic stresses (such as drought, salinity, high temperature and cold) [1]. Among these, drought is one of the most severe abiotic stress factors that limit plant growth and development [2]. Drought stress reduces stomatal conductance, impairs photosynthesis, and induces the accumulation of reactive oxygen species (ROS) [3]. ROS are normal metabolic byproducts in plants and function in oxidative stress response and signal transduction at low levels [4]. However, at elevated concentrations, ROS can damage cellular structures and disrupt normal metabolic processes. Consequently, plants have evolved multiple mechanisms to counteract drought-induced damage. Numerous studies have demonstrated that transcription factors (TFs) play crucial roles in plant responses to various abiotic stresses [5,6,7].

TFs are a class of protein molecules that can bind to specific sequences in DNA, where they either promote or inhibit gene transcription [8], thereby exerting their regulatory functions. In plants, stress-related TFs primarily include five major families: MYB, bZIP, AP2/ERF, WRKY, and NAC [9]. Overexpression of *FvMYB114* and *FvMYB44* from *Fragaria vesca* enhances salt and cold tolerance in transgenic *Arabidopsis thaliana* [10,11]. Similarly, *VvERF63* from grape confers cold stress tolerance in transgenic *A. thaliana* by enhancing photosynthetic capacity and mitigating cellular damage [12].

Among these, WRKY TFs are named after their highly conserved WRKY domain [13]. The N-terminus of the WRKY domain contains a conserved motif, WRKYGQK, while the C-terminus consists of a conserved zinc finger motif [14]. Based on the invariant WRKY motif and varying zinc finger structures, WRKY TFs are categorized into three groups: Group I contains two WRKY domains, while Groups II and III possess only one [15]. Since the first WRKY gene, *SPF1*, was identified in sweet potato [16], members of the WRKY transcription factor family have been continuously discovered in various species. These studies revealed that many WRKY family members are involved in plant responses to abiotic stress [17,18,19]. For instance, *BpWRKY32* in birch enhances salt tolerance by reducing water loss, improving osmotic potential, and decreasing reactive ROS accumulation [20]. Similarly, *HpWRKY85* from St. John’s wort and *BnWRKY49* from ramie enhance drought tolerance in *A. thaliana* [21,22]. In apple and tomato, overexpression of *MdWRKY56* and *SlWRKY17*, respectively, results in reduced malondialdehyde (MDA) and ROS accumulation, increased proline (Pro) content, and greater drought tolerance [23,24].

*Medicago falcata* L. is a perennial leguminous forage grass that harbors important genetic resources for stress resistance, making it a candidate model plant for studying the mechanisms of stress tolerance in legumes [25,26,27]. However, in China, *M. falcata* is primarily distributed in the northwest and northeast, where drought is one of the major factors affecting alfalfa production. In our previous study, we identified *MfWRKY40* as a stress-responsive gene through transcriptomic analysis of drought-stressed *M. falcata* plants and demonstrated that it exhibits higher expression levels in the leaves and roots of *M. falcata* and is significantly induced by drought stress [28]. In this study, we utilized heterologous expression in *A. thaliana* to functionally characterize this gene under both controlled (culture medium) and natural (soil) conditions. Our comprehensive analysis included changes in various components of the ROS scavenging pathway, quantification of physiological parameters (water loss rate, fresh/dry weight), and the expression level of drought-related genes. These findings reveal the molecular mechanism of *MfWRKY40*-mediated drought tolerance in *M. falcata*, establishing a crucial foundation for further exploration of this gene.

## 2. Results

### 2.1. Generation of Transgenic A. thaliana Overexpressing MfWRKY40

**Figure 1:**
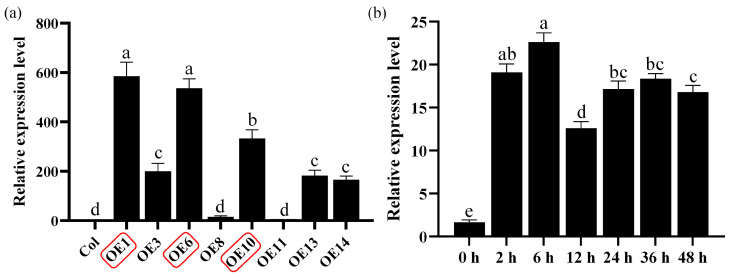
Relative expression levels of the *MfWRKY40* gene in different *Arabidopsis thaliana* lines and analysis of its expression patterns in *Medicago falcata* under mannitol stress. (**a**) Relative expression levels of the *MfWRKY40* gene in wild-type (Col) and different transgenic (OEs) *A. thaliana* lines. (**b**) Relative expression levels of the *MfWRKY40* gene in *M. falcata* under 300 mM mannitol treatment at 0, 2, 6, 12, 24, 36, and 48 h. The transgenic *A. thaliana* lines (OE1, OE6, and OE10) with high expression levels (highlighted in red) were selected for further experiments. All values are expressed as the mean ± standard error (*n* ≥ 3). The different lowercase letters indicate significant differences among various lines (**a**) and significant differences among different time points after 300 mM mannitol treatment (**b**) (*p* < 0.05). Error bars represent the standard error among replicates.

To investigate the functional role of *MfWRKY40* in plants, we constructed a pB_7_WG_2_RS overexpression vector and introduced it into wild-type *A. thaliana* plants (Col) using the floral dip method. After 25 d of normal growth, T_0_ seeds were collected and screened under a fluorescence microscope to select bright red fluorescent seeds. Due to the presence of the RedSeed selection marker, positive transgenic *A. thaliana* lines (OEs) could be easily screened under a fluorescence microscope. Following three consecutive generations of selection, homozygous *A. thaliana* lines were identified. Quantitative real-time PCR (qRT-PCR) analysis showed that *MfWRKY40* expression levels varied among transgenic lines, with all exhibiting significantly higher expression compared to wild-type lines (Figure 1a). Among the transgenic lines, OE1 exhibited the highest *MfWRKY40* expression (584-fold increase relative to wild-type), followed by OE6 (536-fold) and OE10 (333-fold). The remaining lines (OE8, OE11, and OE14) showed basal expression levels comparable to Col (Figure 1a). Based on these results, we selected lines OE1, OE6, and OE10 for further drought tolerance assays.

We performed expression pattern analysis of the *MfWRKY40* gene in *M. falcata*, which revealed that its relative expression levels began to increase 2 h after 300 mM mannitol treatment, peaked at 6 h, and subsequently declined (Figure 1b). These findings demonstrate that *MfWRKY40* expression is induced by mannitol stress.

### 2.2. Response of the Transgenic A. thaliana to Mannitol Treatment

**Figure 2:**
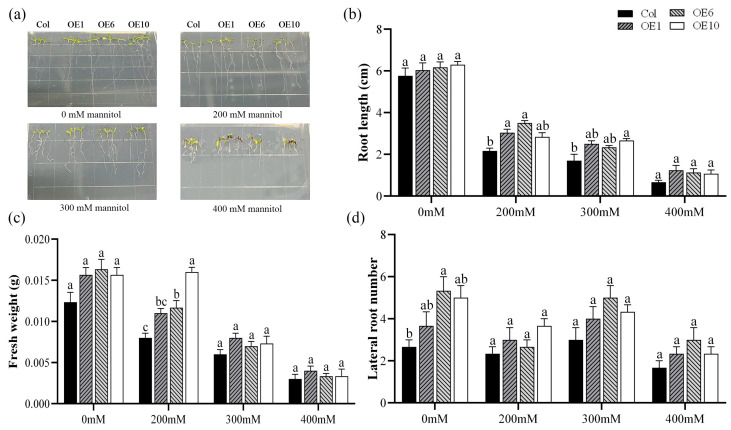
Phenotype comparison of wild-type (Col) and transgenic (OEs) *A. thaliana* lines grown on medium supplied with different concentration of mannitol for 7 d. (**a**) Phenotypic images of *A. thaliana* seedlings. (**b**) Root length. (**c**) Fresh weight. (**d**) Lateral root number. All values are expressed as the mean ± SE (*n* ≥ 15). The different lowercase letters at the same mannitol concentration indicate significant differences among the four lines (*p* < 0.05). Error bars represent the standard error among replicates.

To elucidate the role of *MfWRKY40* in drought stress response, Col and transgenic *A. thaliana* were subjected to stress treatment on media containing 0 mM, 200 mM, 300 mM, and 400 mM mannitol. After 7 d of treatment, root phenotype was photographed and root length, fresh weight, and lateral root number were measured (Figure 2a). The measurements revealed no significant differences in root length among the four lines when grown on normal MS medium (Figure 2b). However, under stress conditions, both Col and transgenic lines displayed progressively reduced primary root length and lateral root number with increasing mannitol concentrations (Figure 2b,d). Fresh weight gradually decreased under increasing stress. Notably, at 200 mM mannitol, transgenic lines exhibited significantly higher fresh weight than Col. Specifically, OE1, OE6, and OE10 exhibited fresh weights of 0.011 g, 0.012 g, and 0.016 g, respectively, compared to 0.008 g in wild-type plants (Figure 2c). These results demonstrate that *MfWRKY40* responds to mannitol-induced stress and enhances *A. thaliana* tolerance to mannitol.

### 2.3. Response of the MfWRKY40 Gene to ABA

**Figure 3:**
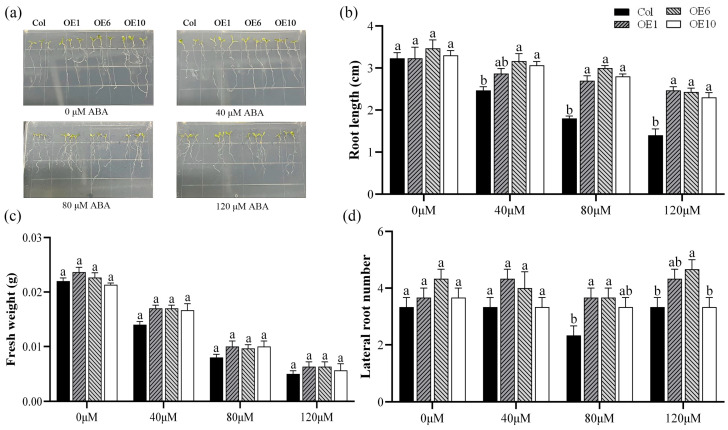
Phenotype comparison of wild-type (Col) and transgenic (OEs) *A. thaliana* lines grown on medium supplied with different concentration of ABA for 7 d. (**a**) Phenotypic images of *A. thaliana* seedlings. (**b**) Root length. (**c**) Fresh weight. (**d**) Lateral root number. All values are expressed as the mean ± SE (*n* ≥ 15). The different lowercase letters at the same ABA concentration indicate significant differences among the four lines (*p* < 0.05). Error bars represent the standard error among replicates.

Abscisic acid (ABA) is a crucial phytohormone that plays an essential role in plant stress response and tolerance. To investigate whether the *MfWRKY40* gene responds to ABA stress, we treated Col and transgenic *A. thaliana* with MS medium containing 0 μM, 40 μM, 80 μM, and 120 μM ABA. Our results demonstrated that after 7 d of cultivation on MS medium without ABA (0 μM), no significant differences were observed in root length, lateral root number, or fresh weight between the transgenic lines and Col (Figure 3a). After 7 d of growth on ABA-supplemented media (40 μM, 80 μM, and 120 μM), all lines showed significant reductions in primary root length, with transgenic *A. thaliana* showing greater root length than Col (Figure 3b). Fresh weight also exhibited a decreasing trend with increasing ABA concentrations, with the fresh weight of Col reaching its lowest value (0.005 g) at 120 μM ABA (Figure 3c,d). These findings strongly indicate that *MfWRKY40* plays a responsive role in ABA-mediated stress regulation.

### 2.4. Phenotypic Analysis of Transgenic A. thaliana

**Figure 4:**
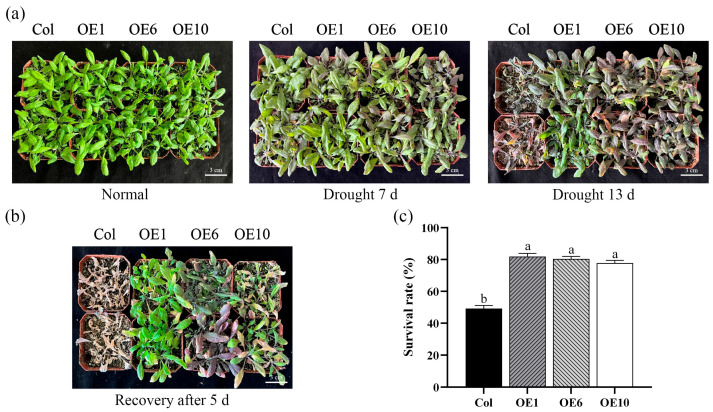
Phenotypes and survival rate of wild-type (Col) and transgenic (OEs) *A. thaliana* under drought stress. (**a**) Phenotypic images of *A. thaliana* under drought stress. Scale bar = 3 cm. (**b**) Phenotypic images of *A. thaliana* after 5 d of rewatering. Scale bar = 3 cm. (**c**) Survival rate after 5 d of rewatering. All values are expressed as the mean ± SE (*n* ≥ 3). The different lowercase letters indicate significant differences among the four lines (*p* < 0.05). Error bars represent the standard error among three replicates.

To further investigate the role of *MfWRKY40* under drought stress, both the Col and transgenic *A. thaliana* were subjected to natural drought stress in soil. Under normal watering conditions, no significant growth differences were observed between Col and transgenic lines (Figure 4a). After 7 d of drought treatment, Col were severely affected, with basal leaves turning to purplish-red, while OE1, OE6, and OE10 exhibited only slight purplish-red discoloration and maintained relatively better growth vigor. Following 13 d of drought stress, wild-type *A. thaliana* stopping growing, while transgenic lines maintained slow growth with partial leaves retaining green color (Figure 4a). Upon rewatering, Col plants exhibited no recovery after 5 d but progressed to severe wilting, whereas transgenic lines developed new green leaves (Figure 4b). The survival rates were 81.8%, 80.3%, and 77.8% for OE1, OE6, and OE10, respectively, compared to only 49.2% for Col (Figure 4c), demonstrating significantly higher survival in transgenic lines. These results indicate that transgenic *A. thaliana* exhibits generally enhanced drought tolerance and superior recovery capacity after rewatering compared to Col.

### 2.5. MfWRKY40 Reduces Water Loss in Transgenic A. thaliana

**Figure 5:**
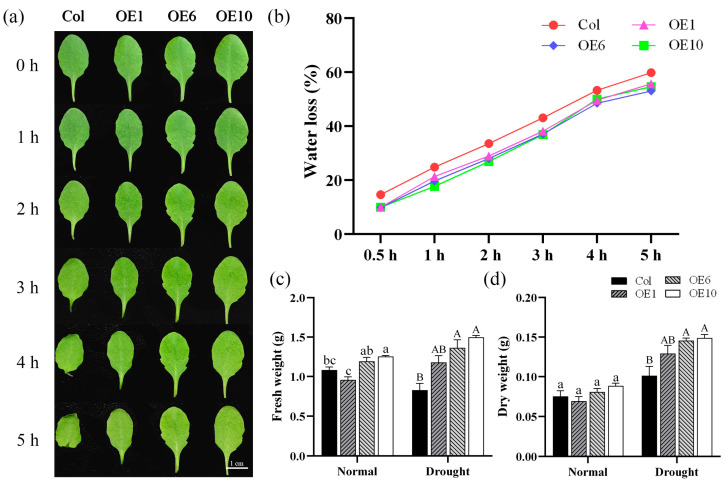
Water loss rate and fresh/dry weight of wild-type (Col) and transgenic (OEs) *A. thaliana*. (**a**) Water loss in detached leaves of *A. thaliana* at 0, 1, 2, 3, 4, and 5 h. Scale bar = 1 cm. (**b**) Water loss rate. (**c**) Fresh weight of *A. thaliana* before drought treatment and after 10 d of treatment. (**d**) Dry weight. All values are expressed as the mean ± SE (*n* ≥ 10). The different lowercase letters indicate significant differences among the four lines before stress treatment (*p* < 0.05), while different uppercase letters indicate significant differences among the four lines after stress treatment (*p* < 0.05). Error bars represent the standard error among replicates.

We also investigated the water loss rates of Col and transgenic *A. thaliana* under normal conditions. Leaves from Col and OEs under normal growth conditions were collected, and phenotypic photographs were recorded at designated time points. It was observed that starting from 1 h, the petioles of Col leaves began to curl, with the curling becoming more pronounced over time. By 4 h, the entire leaf edges started to curl inward, showing obvious signs of dehydration (Figure 5a). Meanwhile, water loss rate measurements revealed that Col exhibited the most rapid water dissipation, reaching 14.6% at 0.5 h, while the transgenic lines showed significantly lower water loss at merely 9.85% (Figure 5b). Col and transgenic lines were subjected to drought stress, and their fresh and dry weights were measured before and after the treatment. The results showed that after stress, the transgenic lines maintained significantly higher fresh and dry weights than Col (Figure 5c,d), indicating that transgenic *A. thaliana* sustained less damage and retained more biomass under drought conditions. Therefore, we concluded that transgenic *A. thaliana* exhibited slower water loss than Col, and that *MfWRKY40* overexpression confers reduced water loss capacity.

### 2.6. Effects of MfWRKY40 on Physiological Indicators

**Figure 6:**
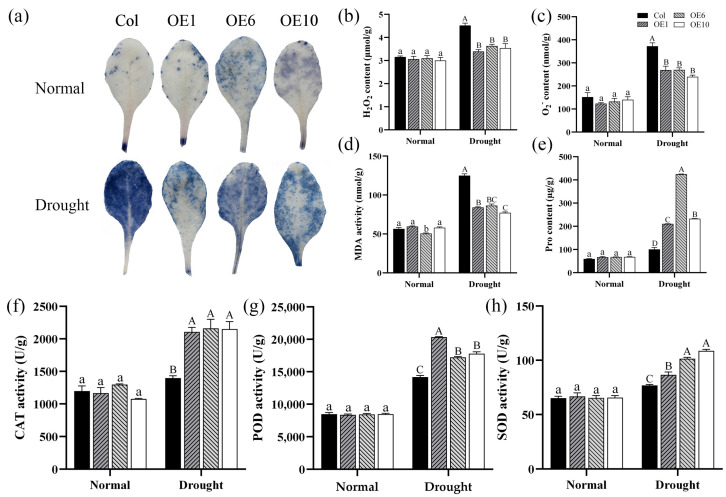
Physiological indices of wild-type (Col) and transgenic (OEs) *A. thaliana* under drought stress. (**a**) NBT staining of *A. thaliana* before drought treatment and after 5 d of drought stress. (**b**) Hydrogen peroxide (H_2_O_2_) content. (**c**) Superoxide anion (O_2_^−^) content. (**d**) Malondialdehyde (MDA) content. (**e**) Proline (Pro) content. (**f**) Catalase (CAT) activity. (**g**) Peroxidase (POD) activity. (**h**) Superoxide dismutase (SOD) activity. All values are expressed as the mean ± SE (*n* ≥ 3). The different lowercase letters indicate significant differences among the four lines before stress treatment (*p* < 0.05), while different uppercase letters indicate significant differences among the four lines after stress treatment (*p* < 0.05). Error bars represent the standard error among replicates.

To investigate the impact of *MfWRKY40* on physiological indicators in *A. thaliana*, we conducted NBT staining and measured the content of hydrogen peroxide (H_2_O_2_) and superoxide anion (O_2_^−^), and MDA, as well as the activity of key antioxidant enzymes. The results showed that before drought stress, no significant differences were observed in the content of these substances between Col and transgenic lines (Figure 6). However, under drought stress, Col accumulated significantly higher levels of H_2_O_2_, O_2_^−^, and MDA compared to transgenic lines. Specifically, O_2_^−^ content reached 372.48 nmol/g and MDA levels increased to 124.95 nmol/g, both markedly higher than in the transgenic line (Figure 6b–d). Additionally, NBT staining revealed more extensive formazan deposition (indicated by larger and darker blue areas) in Col compared to transgenic lines, demonstrating greater oxidative damage in the Col (Figure 6a). Comparative analysis demonstrated that although both the activities of the antioxidant enzymes superoxide dismutase (SOD), catalase (CAT), and peroxidase (POD) and Pro content increased in Col under stress conditions, the magnitude of the increase was significantly smaller than that in transgenic lines (Figure 6e–h). Based on these findings, it can be concluded that the transgenic lines enhance drought tolerance in *A. thaliana* by reducing the accumulation of ROS such as H_2_O_2_ and O_2_^−^ and by increasing the activity of antioxidant enzymes.

### 2.7. Regulation of Enzymes and Drought-Related Genes by MfWRKY40

**Figure 7:**
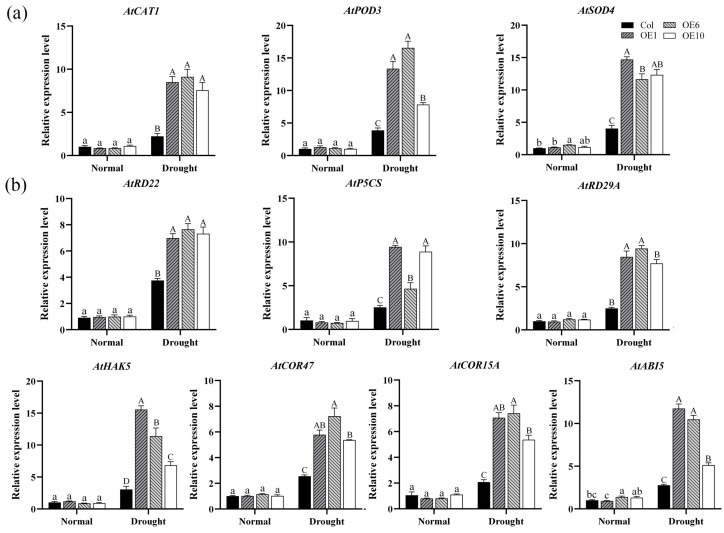
Relative expression levels of enzyme and drought-related genes in wild-type (Col) and transgenic (OEs) *A. thaliana* under drought stress. (**a**) Relative expression levels of enzyme-related genes in *A. thaliana* before drought treatment and after 5 d of drought stress. (**b**) Relative expression levels of drought-related genes. All values are expressed as the mean ± SE (*n* ≥ 3). The different lowercase letters indicate significant differences among the four lines before stress treatment (*p* < 0.05), while different uppercase letters indicate significant differences among the four lines after stress treatment (*p* < 0.05). Error bars represent the standard error among three replicates.

Transcription factors typically exert their roles by regulating the expression of downstream target genes in a coordinated manner. In our study, we analyzed the effect of the *MfWRKY40* gene on the expression levels of antioxidant enzyme genes and drought-related genes. The results demonstrated that under normal conditions, both Col and transgenic lines exhibited low expression levels of *AtPOD3*, *AtSOD4*, and *AtCAT1*. However, after drought stress, their relative expression levels showed differential upregulation patterns. Notably, the expression of *AtPOD3* and *AtSOD4* in transgenic *A. thaliana* exhibited more pronounced changes, while the increase in *AtCAT1* was relatively modest but still significantly higher than Col (Figure 7a). These results suggests that *MfWRKY40* enhances ROS-scavenging capacity through transcriptional upregulation of these three genes, consequently modulating antioxidant activity in transgenic lines.

A similar trend was observed for drought-related genes (*AtHAK5*, *AtCOR47*, *AtCOR15A*, *AtABI5*, *AtRD22*, *AtP5CS*, and *AtRD29A*): under drought stress conditions, overexpression of *MfWRKY40* induced the expression of all seven tested genes, with their expression levels being significantly higher in transgenic lines compared to Col (Figure 7b). These findings demonstrate that *MfWRKY40* modulates the drought stress response in *A. thaliana* by regulating the expression of these key genes.

## 3. Discussion

WRKY TFs are one of the largest families of transcription factors in plants and play a crucial role in plant responses to abiotic stress [29]. Numerous studies have demonstrated that WRKY TFs can enhance stress tolerance in various plants, such as *TaWRKY76*, *TaWRKY2*, and *TaWRKY24* in wheat [30,31,32], *OsWRKY97* in rice [33], and *ZmWRKY25* and *ZmWRKY30* in maize [34,35], which confer stress tolerance through multiple regulatory pathways. In alfalfa, *MsWRKY11* is induced by PEG and ABA, and enhances drought tolerance in alfalfa by reducing leaf stomatal density and improving water use efficiency [36]. Similarly, *MsWRKY42* exhibits the highest expression levels in roots and leaves, with its expression being induced by PEG stress [37]. However, studies on the drought tolerance of the WRKY family have primarily focused on alfalfa, while research on their counterparts in *M. falcata* remains limited. Therefore, in our study, the *MfWRKY40* gene from *M. falcata* was investigated to explore its role in drought tolerance. Using the floral dip method, we introduced *MfWRKY40* into *A. thaliana*, and subsequently treated the transgenic lines with mannitol and ABA. The results showed that under these treatments, *MfWRKY40*-overexpressing *A. thaliana* exhibited significantly better root length performance compared to Col. Notably, under 200 mM treatment, the transgenic lines showed significantly higher fresh weight than wild-type (Figure 2 and Figure 3). Similarly, overexpression of the *Iris germanica* genes *IgWRKY50* and *IgWRKY32*, along with wheat *TaWRKY46*, in *A. thaliana* resulted in transgenic lines exhibiting higher germination rates and longer root lengths on 1/2 MS medium containing mannitol [38,39]. Roots, as the primary sensors of abiotic stress [40,41], develop enhanced growth under drought conditions to facilitate water uptake. The study by Reicosky and Deaton (1979) demonstrated that cultivars with well-developed root systems exhibit enhanced drought tolerance [42]. These results preliminarily suggest that the *MfWRKY40* gene plays a role in root growth and drought tolerance in *A. thaliana*.

Drought stress induces excessive accumulation of reactive ROS in plants, including typical forms such as H_2_O_2_ and O_2_^−^ [43,44]. While high concentrations of ROS can cause oxidative damage, ROS within a certain range also serve as crucial signaling molecules involved in various physiological and biochemical processes [4]. MDA, a primary byproduct of membrane lipid peroxidation, serves as a reliable indicator of oxidative stress severity. Elevated MDA levels directly correlate with the extent of cellular oxidative damage [45]. Antioxidant enzymes such as SOD, CAT, and POD play essential roles in scavenging excess ROS [46], and their activities are indicative of plant stress tolerance under abiotic stress [47,48,49]. Pro, an important osmotic regulator, helps to maintain cellular osmotic balance and protects membrane integrity [13]. Previous studies demonstrated that *ItfWRKY70* from *Ipomoea trifida* increases Pro content, enhances SOD and POD activities, and reduces MDA and H_2_O_2_ levels [50]. In parallel, *SlWRKY8* from *Solanum lycopersicum* was shown to regulate ROS scavenging pathways, conferring drought tolerance through enhanced ROS elimination [51]. In our study, under drought stress, *MfWRKY40*-overexpressing *A. thaliana* lines exhibited significantly higher antioxidant enzyme activities and Pro content compared to wild-type, along with lower levels of MDA, H_2_O_2_, and O_2_^−^ (Figure 6). Additionally, we quantified the expression levels of key antioxidant enzyme-related genes, including *AtPOD3*, *AtSOD4*, and *AtCAT1*. The results consistently showed that the gene expression levels in transgenic *A. thaliana* were significantly higher than those in wild-type under drought stress (Figure 7a). These findings demonstrate that *MfWRKY40* enhances drought tolerance by participating in ROS scavenging pathways.

The plant’s response to abiotic stress is a complex physiological process orchestrated by multiple genes functioning synergistically. WRKY TFs recognize and bind to W-box cis-elements in drought-responsive gene promoters [51], thereby regulating their expression and enhancing plant drought tolerance. *P5CS1* is a key gene in Pro biosynthesis, which modulates plant adaptive responses to abiotic stress through Pro accumulation [52]. *RD29B* and *RD29A* are dehydration-inducible genes in *A. thaliana* that encode hydrophilic proteins [53]. The *RD29A* gene product mitigates damage caused by various abiotic stresses including drought, salinity, and cold [54]. Other drought-responsive genes exhibit similar functions, where upregulated expression enhances stress tolerance. Research demonstrates that WRKY TFs regulate drought-responsive gene networks through coordinated modulation of multiple signaling pathways, thereby affecting plant drought adaptation. *PheWRKY86* from *Phyllostachys edulis* is induced by both drought and ABA treatments and enhances drought tolerance by upregulating *NCED1* expression [55]. The *MbWRKY53* gene from apple improves cold and drought adaptation in transgenic *A. thaliana* by modulating the expression of *SOS1*, *COR47*, *DREB2A*, *P5CS1*, *COR6.6*, and *RD29b* [13]. Similarly, overexpression of *MbWRKY40* in apple alters the expression levels of *AtKIN1*, *AtDREB2A*, *AtRD29A*, *AtERD10*, *AtCOR47A*, and *AtRD29B* in *A. thaliana*, thereby enhancing stress tolerance [56]. In our study, the drought-related genes *AtABI5*, *AtCOR15A*, *AtCOR47*, *AtHAK5*, *AtP5CS*, *AtRD22*, and *AtRD29A* were similarly regulated by *MfWRKY40*, exhibiting higher expression levels in transgenic *A. thaliana* (Figure 7b). This suggests that *MfWRKY40* may specifically bind to the W-box elements of these genes to enhance drought tolerance in *A. thaliana*.

## 4. Materials and Methods

### 4.1. Plant Materials

The seeds of *M. falcata* were provided by the College of Grassland Science, Xinjiang Agricultural University (Urumqi, China), while the *A. thaliana* ecotype Columbia-0 (Col-0) seeds were supplied by the Forage Germplasm Resources Conservation and Utilization Innovation Team at the Institute of Animal Sciences, Chinese Academy of Agricultural Sciences (Beijing, China).

Seven-day-old *M. falcata* seedlings were transferred to hydroponic boxes containing Hoagland’s nutrient solution [36], with the solution replaced every two days. After 10 d of cultivation, seedlings were subjected to 300 mM mannitol treatment. Leaf samples were collected at 0, 2, 6, 12, 24, 36, and 48 h after treatment, immediately frozen in liquid nitrogen, and stored at −80 °C for subsequent analysis of *MfWRKY40* relative expression levels.

### 4.2. Cloning of the MfWRKY40 Gene and Construction of the Overexpression Vector

Fresh leaves of *M. falcata* were collected, and total RNA was extracted using the Eastep^®^ Super Total RNA Extraction Kit (LS1040, Promega, Shanghai, China). The extracted RNA was then reverse-transcribed into cDNA using the 5× FastKing-RT Super Mix kit (KR118, Tiangen, Beijing, China), with reaction conditions and master mix compositions detailed in Tables S2 and S3. Gene-specific primers were designed using GeneRunner software (version 5.1.06, Gennady A. Peskin, Moscow, Russia), and the sequences are listed in Table S1. PCR amplification was performed using high-fidelity KOD polymerase (Toyobo, Osaka, Japan) with cDNA as the template, following the reaction system described in Table S4 and the thermal cycling program outlined in Table S5. The amplified product was gel-purified, and the recovered DNA fragment was ligated into the pENTR entry vector (Table S6). The reaction mixture was transformed into DH5α competent *E. coli* cells (Biomed Gene, Beijing, China). Positive clones were selected on Kanamycin (Kan)-containing medium, and the plasmid DNA was extracted. The *MfWRKY40* was subcloned from the pENTR vector into the pB_7_WG_2_RS overexpression vector through the LR reaction. The LR reaction was performed under the conditions specified in Table S7, with incubation at 25 °C for 1 h, followed by treatment with Proteinase K at 37 °C for 10 min. The pB_7_WG_2_RS overexpression vector was a modified version constructed by cloning the RedSeed selection marker (pNAP::DsRed) from pKGW-RedSeed into the pB_7_WG_2_ vector [57], using XbaI and KpnI restriction sites. This vector was provided by the Forage Germplasm Resources Conservation and Utilization Innovation Team at the Institute of Animal Sciences, Chinese Academy of Agricultural Sciences (Beijing, China). The reaction products were directly transformed into DH5α competent *E. coli* cells (Biomed Gene, Beijing, China) and cultured on a medium containing spectinomycin (Spec) tolerance. Single colonies were screened and verified by PCR analysis. Colonies showing clear bands of the expected size were then expanded in large-scale culture. Plasmids were extracted from these cultures and further verified by conventional PCR using gene-specific primers to confirm construct integrity. If the plasmid PCR shows a bright band of the expected size, it indicates successful construction of the overexpression vector.

The successfully constructed pB_7_WG_2_RS overexpression vector was introduced into *Agrobacterium tumefaciens* GV3101 (Biomed Gene, Beijing, China) culture. The transformed bacteria were grown in LB medium supplemented with kanamycin and rifampicin, and the OD value was adjusted to 0.6–0.8 by centrifugation and resuspension in buffer. The resulting *Agrobacterium* suspension was then ready for subsequent *A. thaliana* transformation experiments.

### 4.3. Generation of Transgenic A. thaliana

When *A. thaliana* plants initiated bolting and floral bud formation, newly emerged inflorescences were pruned promptly to promote subsequent flowering. After flowering, siliques were excised, and the plants were watered adequately in preparation for inoculation and infection the following day. T_0_-generation *A. thaliana* seeds were obtained using the floral dip method [57]. Transgenic seeds displaying red fluorescence were selected under a fluorescence microscope (Leica M205 FA, Wetzlar, Germany) [58], then cultivated to maturity. Progeny seeds were repeatedly screened until homozygous T_3_ transgenic *A. thaliana* lines were obtained.

### 4.4. RNA Extraction and Quantitative Real-Time PCR (qRT-PCR)

The samples were flash-frozen in liquid nitrogen for 30 s, then ground into a fine powder. This freeze–grinding cycle was repeated at least three times. Total RNA was subsequently extracted following the protocol in Section 4.2, followed by cDNA synthesis. Using cDNA as the template and the *A. thaliana AtActin*-qPCR-F/R primers as the reference gene [59], the relative expression levels of enzyme-related genes (*AtPOD3*, *AtSOD4*, and *AtCAT1*) and drought-related genes (*AtHAK5*, *AtCOR47*, *AtCOR15A*, *AtABI5*, *AtRD22*, *AtP5CS*, and *AtRD29A*) were quantified by qRT-PCR with the SuperReal PreMix Plus kit (FP205, Tiangen, Beijing, China). Primer sequences are listed in Table S1, while reaction components and cycling conditions are detailed in Supplementary Tables S8 and S9. Three biological replicates were performed, with each biological replicate including three technical replicates. Finally, the experimental data were analyzed, and relative gene expression levels were calculated using the 2^−ΔΔCt^ method [60].

### 4.5. Stress Treatment of Transgenic A. thaliana

By measuring the expression levels of the *MfWRKY40* gene in different transgenic lines (Figure 1a), three *A. thaliana* lines with high expression were selected for further study. For the mannitol and ABA treatment experiment, seeds of Col and transgenic *A. thaliana* were surface-sterilized and evenly plated on 1/2 MS medium, followed by cold stratification at 4 °C in complete darkness for 3 d before being transferred to a plant growth chamber. After 4 d of cultivation on 1/2 MS medium at 22 °C under a 16-hour light/8-hour dark photoperiod with a light intensity of 400 μmol·m^−2^·s^−1^, the seedlings were transferred to MS medium containing different concentrations of mannitol (0, 200, 300, and 400 mM) or ABA (0, 40, 80, and 120 μM) for stress treatment. On the 7th day, the Col and transgenic lines were photographed, and parameters including root length, lateral root number, and fresh weight were recorded.

For the drought experiment, Col and transgenic *A. thaliana* seeds were vernalized in a 4 °C refrigerator in the dark for 3 d, then cultured in a light incubator for 7 d until two small leaves emerged. The *A. thaliana* seedlings were transplanted into small pots (6 cm diameter) containing a mixture of nutrient soil, vermiculite, and soil conditioner (3:1:1 ratio), with 9 plants per pot, and grown in a light-controlled chamber. After 30 d of cultivation, the plants were subjected to stress treatment by withholding water to simulate natural drought. The phenotypes of each line were observed under drought conditions. When most leaves turned purplish-red with yellow edges, rehydration was performed, and the phenotype were further observed. Photographs were taken before drought stress, as well as at 7 d, 13 d of stress treatment, and 5 d after rehydration. Survival rates were recorded before stress treatment and after rehydration.

### 4.6. Determination of Water Loss Rate and Fresh/Dry Weight

For wild-type and transgenic *A. thaliana* plants grown under normal conditions, detached leaves were sampled and photographed at 0, 1, 2, 3, 4, and 5 h to observe phenotypic changes between wild-type and transgenic lines. Concurrently, 0.5 g leaf samples from four different lines were weighed at 0.5, 1, 2, 3, 4, and 5 h to calculate the water loss rate, thereby comparing water dissipation between wild-type and transgenic *A. thaliana* [61]. Prior to drought stress and after 10 d of stress treatment, all *A. thaliana* lines (9 plants per pot) were harvested to measure fresh weight. The samples were then placed in paper envelopes, dried at 105 °C for 30 min, and further oven-dried at 80 °C until constant weight was achieved for dry weight.

### 4.7. NBT Staining and Determination of Physiological Indices

Leaf samples were collected from identical positions on both wild-type and transgenic *A. thaliana* lines before drought stress and after 5 d of stress treatment. One portion of the leaves was stained using the NBT Staining Kit (G4816, Solarbio, Beijing, China), while the other portion was homogenized in either the specified extraction buffer or acetone, followed by centrifugation at 4 °C for 10 min. The resulting supernatant was collected for subsequent physiological assays. The activities of the three antioxidant enzymes were determined using the SOD Assay Kit (YH1202, Angle Gene, Nanjing, China), POD Assay Kit (YH1210, Angle Gene, Nanjing, China), and CAT Assay Kit (YH1208, Angle Gene, Nanjing, China), respectively. Additionally, the contents of MDA, Pro, O_2_^−^, and H_2_O_2_ were determined using the MDA Assay Kit (YH1217, Angle Gene, Nanjing, China), Pro Assay Kit (YH1231, Angle Gene, Nanjing, China), O_2_^−^ Assay Kit (G0116F, Grace Biotechnology, Suzhou, China), and H_2_O_2_ Assay Kit (G0112F, Grace Biotechnology, Suzhou, China).

### 4.8. Data Analysis

Data organization was performed using Microsoft Excel (Version 2016, Redmond, WA, USA). Statistical significance was analyzed using IBM SPSS software (Version 25, Armonk, NY, USA). One-way ANOVA followed by Tukey’s Honestly Significant Difference (HSD) test was applied, with the significance level set at *p* < 0.05. All values are presented as the mean ± standard error (SE). The different lowercase letters indicate significant differences among the four lines before stress treatment (*p* < 0.05), while different uppercase letters indicate significant differences among the four lines after stress treatment (*p* < 0.05). Figures were generated using GraphPad Prism (version 9, San Diego, CA, USA). All experiments were conducted with three biological replicates and three technical replicates.

## 5. Conclusions

**Figure 8:**
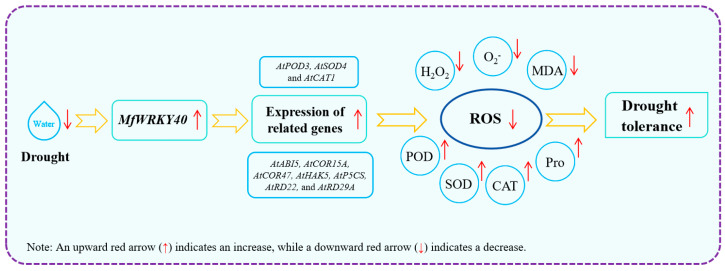
Regulatory model of *MfWRKY40* gene in *A. thaliana* under drought stress.

In this study, Col and transgenic *A. thaliana* were first treated with stress media (mannitol and ABA), revealing that transgenic lines exhibited superior root length and fresh weight compared to Col under identical conditions. Subsequent natural drought treatment further demonstrated enhanced recovery capacity in overexpression lines. Analysis revealed significantly higher water loss rates in wild-type compared to transgenic lines, accompanied by reduced biomass accumulation (both fresh and dry weight) under stress conditions. Meanwhile, *MfWRKY40*-overexpressing lines displayed higher antioxidant enzyme activities and Pro content, along with reduced levels of H_2_O_2_, O_2_^−^, and MDA. Additionally, the expression of antioxidant enzyme genes (*AtPOD3*, *AtSOD4*, and *AtCAT1*) and drought-responsive genes (*AtABI5*, *AtCOR15A*, *AtCOR47*, *AtHAK5*, *AtP5CS*, *AtRD22*, and *AtRD29A*) was induced by *MfWRKY40* in the transgenic lines. Our findings demonstrate that *MfWRKY40* acts as a positive regulator of drought tolerance in *A. thaliana* by enhancing the activities of antioxidant enzymes (POD, SOD, and CAT) while reducing H_2_O_2_, O_2_^−^, and MDA, thereby promoting ROS scavenging (Figure 8). In subsequent studies, we plan to generate transgenic *Medicago sativa* (since the *M. falcata* transformation system is not yet fully established) to further comprehensively evaluate the functional role of *MfWRKY40* in drought stress responses, thereby providing a foundation for molecular breeding in alfalfa.

## Data Availability

The data presented in this study are available in the article or the Supplementary Materials.

## Supplementary Materials

The following supporting information can be downloaded at https://www.mdpi.com/article/10.3390/ijms26178495/s1.
