# Supplementary material for: MfWRKY40 Positively Regulates Drought Tolerance in Arabidopsis thaliana by Scavenging Reactive Oxygen Species
Source: Int J Mol Sci. 2025 Sep 1;26(17):8495. doi: 10.3390/ijms26178495 (PMC12429208; doi:10.3390/ijms26178495)
Supplement: Supplementary file 1 [file ijms-26-08495-s001.zip › ijms-3787099-supplementary.pdf]

Table S1 All primer sequences used in PCR in this experiment

| Prime | Forward primer/ Reverse primer (5′-3′) |
|---|---|
| q*Atactin*-F | TATGAATTACCCGATGGGCAAG |
| q*Atactin*-R | TGGAACAAGACTTCTGGGCAT |
| q*MfWRKY40*-F | TCTCAACGGAGCCGACAAA |
| q*MfWRKY40*-R | TGCGGAAGAAGGTGCTTTCT |
| *Atactin*-F | CGCCATCCAAGCTGTTCTC |
| *Atactin*-R | TCACGTCCAGCAAGGTCAAG |
| q*MfUBL-2a*-F | CCAAACCCAAACTCACCAG |
| q*MfUBL-2a*-R | AGCAGTCCAACTCTGCTCAAC |
| *AtRD22*-F | ACTTGGTAAATATCACGTCAGGGCT |
| *AtRD22*-R | CTGAGGTGTTCTTGTGGCATACC |
| *AtP5CS*-F | GAGCAATGGAGTCACTTTGTATGG |
| *AtP5CS*-R | TTCCTCTCATTATCCATCTCGTTG |
| *AtCOR15A*-F | CAGTTCGTCGTCGTTTCT |
| *AtCOR15A*-R | CCAATGTATCTGCGGTTT |
| *AtHAK5*-F | TGCATCACTGGGACGGAGGCA |
| *AtHAK5*-R | GTACGCAGCCTGCCCGCAAT |
| *AtABI5*-F | AGAGGGATAGCGAACGAGTCTAGTC |
| *AtABI5*-R | GTTCGGGTTTGGATTAGGTTTAGG |
| *AtRD29A*-F | GATATCGACAAGGATGTGCCG |
| *AtRD29A*-R | GTATCCAGGTCTTCCCTTCGC |
| *AtPOD3*-F | CCAATCCGGAAACGGAAGTC |
| *AtPOD3*-R | TCTGCATACTTCTTGACGAG |
| *AtSOD4*-F | GAAGAACCTTGCTCCTTCCAG |
| *AtSOD4*-R | GATTGGCAGTTGTGTCAACAAC |
| *AtCAT1*-F | GTCCTGGGATTCAGACAGGC |
| *AtCAT1*-R | GGCCTCACGTTAAGACGAGT |
| *AtP5CS1*-F | AGGGAAAGTTCCAGAAAG |
| *AtP5CS1*-R | CATAACTAAGCGAGCCAC |
| *MfWRKY40*-F | CACCATGGATTTTTCATCATCATGGAT |
| *MfWRKY40*-R | CTAATTTTGGTGTAACATTTTTCC |

Table S2 Reaction system for the synthesis of the first strand of reverse transcribed cDNA

| Components | Volume |
|---|---|
| 5×FastKing-RT SuperMix | 2 μl |
| Total RNA | 1 μg |
| RNase-Free dd$H_2O$ | To 10 μl |

Table S3 Reaction program for the synthesis of the first strand of reverse transcribed cDNA

| Reaction temperature (℃) | Reaction time (min) | Description |
|---|---|---|
| 42 | 15 | Eliminate the genome and reverse transcription reaction |
| 95 | 3 | Enzyme deactivation process |

Table S4 The PCR reaction system

| Components | Volume (μl) |
|---|---|
| 10×KOD buffer | 2.5 |
| cDNA Template | 2.5 |
| $MgSO_4$ | 1 |
| dNTP mixture（each 10 mM） | 2.5 |
| F（10 μM） | 1 |
| R（10 μM） | 1 |
| KOD Plμs enzyme | 0.5 |
| $ddH_2O$ | 14 |
| Total Volume | 25 |

Table S5 The PCR reaction program

| Step | Temperature (℃) | Time (s) | Recycle |
|---|---|---|---|
| 1 | 94 | 180 | 1 |
| | 94 | 30 | |
| 2 | 58 | 30 | 34 |
| | 68 | 90 | |
| 3 | 68 | 300 | 1 |

Table S6 P-ENTR system for constructing overexpression vector

| Component | Volume (μl) |
|---|---|
| Target gene | 3.2 |
| pENTR vector | 0.4 |
| 10×Enhance | 0.4 |
| Total volume | 4 |

Table S7 LR system for constructing overexpression vectors

| Component | Volume (μl) |
|---|---|
| Target gene | 1 |
| $pB_7WG_2RS$ vector | 1 |
| LR enzyme | 0.5 |
| Protein K | 0.25 |
| Total volume | 2.75 |

Table S8 The qRT-PCR reaction system

| Composition | Usage quantity（μl） |
|---|---|
| 2× Supernal PreMix Plus | 10 |
| Primer-qF（10 μM） | 0.6 |
| Primer-qR（10 μM） | 0.6 |
| cDNA | 1.5 |
| 50× ROX Reference Dye | 0.4 |
| dd $H_2O$ | to 20 |

Table S9 qRT-PCR reaction procedure

| Stage | Recycle | Temperature (℃) | Time |
|---|---|---|---|
| premutability | 1× | 95 | 15 min |
| | | 95 | 10 s |
| PCR reaction | 40× | 60 | 20 s |
| | | 72 | 30 s |
